# The Effect of Antibiotics on Planktonic Cells and Biofilm Formation Ability of Collected *Arcobacter*-like Strains and Strains Isolated within the Czech Republic

**DOI:** 10.3390/antibiotics11010087

**Published:** 2022-01-11

**Authors:** Karolína Švarcová, Marcela Pejchalová, David Šilha

**Affiliations:** Department of Biological and Biochemical Sciences, Faculty of Chemical Technology, University of Pardubice, Studentská 573, 53210 Pardubice, Czech Republic; Karolina.Svarcova@student.upce.cz (K.Š.); toskan@centrum.cz (M.P.)

**Keywords:** antibiotics, antibiotic resistance, MIC, biofilm, *Arcobacter*-like, *Aliarcobacter butzleri*, *Aliarcobacter cryaerophilus*

## Abstract

The purpose of this study was to test the in vitro effects of ampicillin, ciprofloxacin, clindamycin, erythromycin, gentamicin, and tetracycline on planktonic cells of *Arcobacter*-like microorganisms and on their biofilm formation ability. The minimum inhibitory concentrations (MICs) were determined by the microdilution method. Further, biofilm formation ability in the presence of various concentrations of antibiotics was evaluated by a modified Christensen method. Most of the 60 strains exhibited high susceptibility to gentamicin (98.3%), ciprofloxacin (95.0%), and erythromycin (100.0%). High level of resistance was observed to clindamycin and tetracycline with MIC_50_ and MIC_90_ in range of 4–32 mg/L and 32–128 mg/L, respectively. Combined resistance to both clindamycin and tetracycline was found in 38.3% of tested strains. In general, higher biofilm formation was observed especially at lower concentrations of antibiotics (0.13–2 mg/L). However, a significant decrease in biofilm formation ability of *Pseudarcobacter defluvii* LMG 25694 was exhibited with ampicillin and clindamycin at concentrations above 32 or 8 mg/L, respectively. Biofilm formation represents a potential danger of infection and also a risk to human health, in particular due to antimicrobial-resistant strains and the ability to form a biofilm structure at a concentration that is approximately the MIC determined for planktonic cells.

## 1. Introduction

The genera *Campylobacter*, *Arcobacter*, and *Sulfurospirillum* belonged to the class *Epsilonproteobacteria* and the family *Campylobacteraceae* due to their close genotypic and phenotypic similarities [1,2]. However, a reclassification of the class *Epsilonproteobacteria* to the class *Campylobacteria* has been proposed according to previous results. Based on these findings, the genus *Arcobacter* should be reclassified into the family *Arcobacteraceae* and the class *Campylobacteria* [3,4,5]. *Arcobacter*-like microorganisms currently contain 29 recognized species; however, this number is rapidly increasing [3,6]. These microorganisms are Gram-negative bacteria, the rod cells can be slightly curved [7]. These bacteria typically grow in a microaerophilic environment, but some species are able to grow under aerobic or anaerobic conditions [8]. Arcobacters are widespread, the typical occurrence is in foodstuffs of animal origin, water, and various locations in the environment [9,10].

Moreover, the number of diseases caused by arcobacters is increasing; however, their mechanisms of action and the dependence of their pathogenicity on virulence factors have not been sufficiently explained and understood [10,11,12]. Several genes are considered to be important for virulence, due to their homologs with virulence genes in other pathogenic bacteria [13]. The virulence factors also include the ability to adhere to various surfaces and biofilm formation [14,15,16]. These bacteria are implicated as causative agents of diarrhea, mastitis, and abortion in animals, while causing bacteremia, endocarditis, peritonitis, gastroenteritis, and diarrhea in humans. Manifestations of the disease are often similar to those described for campylobacteriosis [17]. The association of arcobacters with human disease has been demonstrated for *Aliarcobacter butzleri*, *Aliarcobacter cryaerophilus*, *Aliarcobacter skirrowii*, and *Aliarcobacter thereius* [18].

Infections caused by *Arcobacter*-like microorganisms are mainly self-limiting. Antimicrobial therapy is usually not required, although in severe cases antibiotic treatment is necessary [19,20]. Arcobacters are generally sensitive to fluoroquinolones, tetracyclines, and aminoglycosides. These antibiotics are recommended for use in human and veterinary medicine [21]. However, a significant level of resistance to clindamycin, azithromycin, ciprofloxacin, cefoperazone, and other antimicrobials has been reported previously for arcobacters [19,22,23].

The subject of this study was to obtain data regarding the effect of selected antibiotics on planktonic cells for many *Arcobacter*-like strains (determination of minimum inhibitory concentration values). The paper is also focused on monitoring the effect of selected antibiotics on biofilm formation in collection strains of arcobacters and strains isolated within the Czech Republic. To our knowledge, previous studies are limited and have not shown in more detail the effect of antibiotics on biofilm formation in these potentially pathogenic bacteria. The results of this study may therefore add to previous knowledge.

## 2. Results

### 2.1. Antibiotic Susceptibility of Arcobacter-like Strains

A total of 60 *Arcobacter*-like strains, mostly isolated within the Czech Republic, were evaluated for resistance or susceptibility to many selected antimicrobials used in clinical practice. Expressions of the antimicrobial effect of individual antibiotics are presented in Table 1. In all cases, the MIC and MBC values were identical. For some antibiotics, the MIC_50_ and MIC_90_ values were almost identical (maximum two-fold difference); however, a significant difference between these concentrations was observed for ampicillin (8 and 64 mg/L) and tetracycline (4 and 64 mg/L) for *Aliarcobacter* (*A.*) *butzleri* strains. The determined MIC_50_ and MIC_90_ of ampicillin were 8 mg/L and 64 mg/L, and the recorded MIC_50_ and MIC_90_ of tetracycline were 4 mg/L and 64 mg/L for *A. butzleri* strains, respectively. Similarly, the determined MIC_50_/MIC_90_ of ampicillin were 8 mg/L and 128 mg/L, and these concentrations for ciprofloxacin were 0.13 mg/L and 64 mg/L for *A. cryaerophilus* strains, respectively.

As shown in Table 1, 47/60 (78.3%) of strains were sensitive to ampicillin, 57/60 (95.0%) to ciprofloxacin, 7/60 (11.7%) to clindamycin, 60/60 (100.0%) to erythromycin, 59/60 (98.3%) to gentamicin and 24/60 (40.0%) were sensitive to tetracycline based on the breakpoints defined by the Clinical Laboratory Standards Institute established for *C. coli* and the *Enterobacteriaceae* family [24]. Our results indicate that arcobacters are mostly sensitive to macrolides, aminoglycosides, and quinolones. Erythromycin (MIC_50_/MIC_90_ of 2 mg/L and 4 mg/L), gentamicin (*A. butzleri* strains—MIC_50_/MIC_90_ of 0.25 mg/L and 1 mg/L; *A. cryaerophilus* strains—MIC_50_/MIC_90_ of 0.5 mg/L and 1 mg/L), and ciprofloxacin (*A. butzleri* strains—MIC_50_/MIC_90_ 0.06 mg/L and 0.13 mg/L) were evaluated to be the most effective antibiotics against *Arcobacter*-like strains.

On the other hand, considerable resistance of strains has been noted for linkosamides. Most of the *Arcobacter*-like strains (88.3%) were resistant to clindamycin. *Arcobacter*-like strains were also more resistant to β-lactam antibiotics (ampicillin; MIC_50_/MIC_90_ values of 8 mg/L and 64 mg/L for *A. butzleri* strains and MIC_50_/MIC_90_ values of 8 mg/L and 128 mg/L for *A. cryaerophilus* strains) and tetracyclines (tetracycline; MIC_50_/MIC_90_ values of 4 mg/L and 64 mg/L). The observed resistance levels were 21.7% for ampicillin and 5.0% for ciprofloxacin, 88.3% for clindamycin, 1.7% for gentamicin and 60.0% for tetracycline. None of the studied strains exhibited resistance to erythromycin.

Furthermore, a high level of resistance to several antibiotics was observed (Table 2). Specifically, *A. butzleri* LMG 10,828 was observed to exhibit resistance to four antibiotics included in our study and can be considered to be a multi-resistant strain. Similarly, *A. butzleri* UPa 2013/12 was evaluated as resistant to ampicillin, ciprofloxacin, clindamycin, and tetracycline (see Table 2). Nine strains (15.0%) were even evaluated as resistant to the combination of three antibiotics—in seven cases it was a combination of ampicillin, clindamycin, and tetracycline, and in two cases a combination of ciprofloxacin, clindamycin, and tetracycline. Combined resistance to both clindamycin and tetracycline was observed in 23/60 (38.3%) of strains.

### 2.2. Effect of Antibiotic Presence on Biofilm Formation Ability of Arcobacter-like Strains

**Figure 1:**
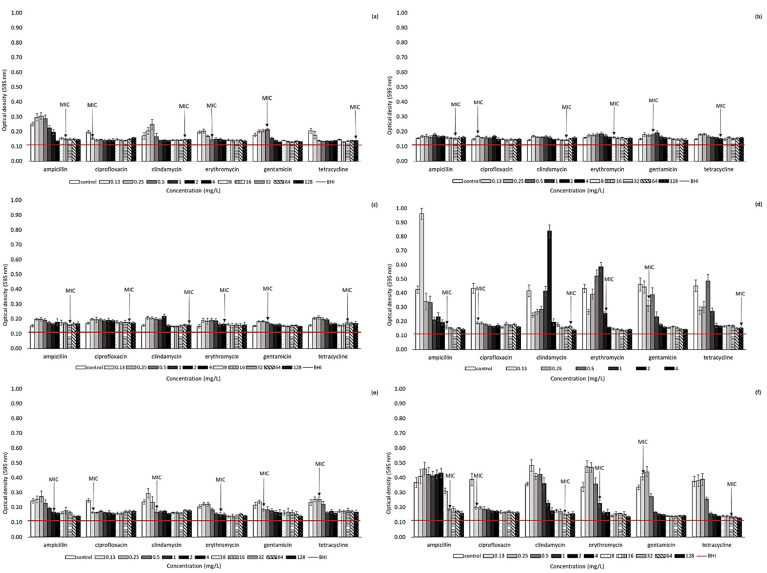
Biofilm formation in the presence of antibiotics. (**a**) *Aliarcobacter butzleri* CCUG 30484; (**b**) *Aliarcobacter butzleri* UPa 2013/6 (isolated from food); (**c**) *Aliarcobacter butzleri* UPa 2013/9 (isolated from wastewater sample); (**d**) *Aliarcobacter butzleri* UPa 2013/30 (isolated from food); (**e**) *Aliarcobacter butzleri* UPa 2015/13 (isolated from food); (**f**) *Aliarcobacter butzleri* UPa 2015/14 (isolated from food). The horizontal line represents the influence of BHI broth (values under horizontal line—biofilm-negative; values above line—biofilm-positive).

**Figure 2:**
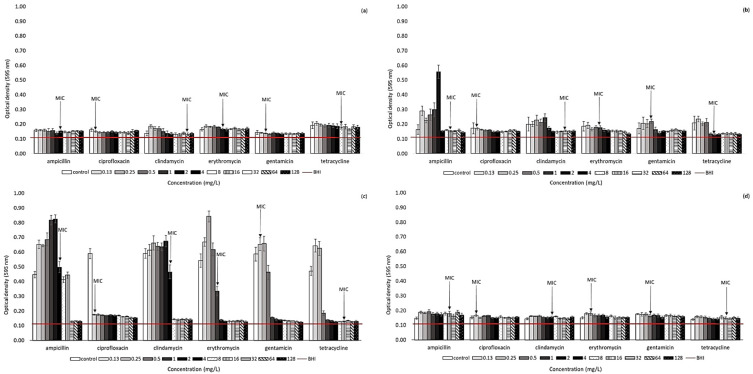
Biofilm formation in the presence of antibiotics. (**a**) *Aliarcobacter cryaerophilus* CCM 7050; (**b**) *Aliarcobacter cryaerophilus* UPa 2013/13 (isolated from water); (**c**) *Pseudarcobacter defluvii* LMG 25694; (**d**) *Aliarcobacter skirrowii* LMG 6621. The horizontal line represents the influence of BHI broth (values under horizontal line—biofilm-negative; values above line—biofilm-positive).

The biofilm-forming ability of 10 *Arcobacter*-like strains was monitored in the presence of various concentrations of antibiotics (Figure 1 and Figure 2). This testing was conducted on selected strains to specifically include *A. butzleri* and *A. cryaerophilus* strains (the most represented of the strains included in this study). Furthermore, the effect of selected antibiotics was monitored for *Pseudarcobacter defluvii* LMG 25,694 and *A. skirrowii* LMG 6621 as a strain with high biofilm activity and one more frequently isolated from various samples, respectively. According to our results, biofilm formation ability can be observed in all the strains included in our study (OD_595_ without the effect of antibiotics is over 0.120). However, the individual strains differ in the intensity of biofilm formation. Overall, the highest primary biofilm formation (without the presence of antibiotics) was observed for *A. butzleri* UPa 2013/30 and *Pseudarcobacter defluvii* LMG 25694, with measured OD_595_ ranging from 0.415–0.461 and 0.446–0.590, respectively.

In general, higher biofilm formation among *Arcobacter*-like strains was observed especially at lower concentrations (0.13–2 mg/L) of the tested antibiotics, e.g., the strain *A. butzleri* CCUG 30,484 exhibited increased biofilm formation ability, especially at lower antibiotic concentrations (c_ATB_ below 0.5–1 mg/L). Further, higher antibiotic concentrations lead to a decrease in biofilm formation. Otherwise, the strain *Pseudarcobacter defluvii* LMG 25,694 only exhibited a significant decrease in biofilm formation at ampicillin concentrations at or above 32 mg/L and clindamycin concentration at or above 8 mg/L. However, a concentration of 4 mg/L has already been evaluated as the MIC of ampicillin and clindamycin for planktonic cells of this strain. Similarly, an increased biofilm production of *A. butzleri* strain UPa 2013/30 was recorded in the presence of many monitored antibiotics (ampicillin, clindamycin, erythromycin, gentamicin, and tetracycline) up to a concentration of 2–4 mg/L.

A similar trend was observed for other strains as well. This fact can be explained by the stressful conditions of a low concentration of antibiotics, at which bacteria could not survive any longer in their planktonic form and immediately formed a biofilm structure. Thus, at higher antibiotic concentrations, biofilm activity was significantly suppressed, as shown in Figure 1 and Figure 2. Generally, the results of this study clearly indicate that the concentration of antibiotics below the MIC values could significantly support the biofilm formation of these bacteria. Accordingly, a considerable decrease in biofilm formation was observed, e.g., for *A. butzleri* CCUG 30,484 in the environment of ampicillin at a concentration above 2 mg/L and for *A. cryaerophilus* UPa 2013/13 at a concentration of clindamycin above 2 mg/L. Further, the biofilm formation of *A. butzleri* UPa 2015/14 was significantly reduced in the presence of ampicillin at a concentration of up to at least 16 mg/L, which corresponds to the MIC concentration determined for planktonic cells. A considerable inhibition of biofilm formation activity was observed with the presence of ciprofloxacin, e.g., for *A. butzleri* CCUG 30484, *Pseudarcobacter defluvii* LMG 25694, *A. butzleri* UPa 2013/30, *A. butzleri* UPa 2015/13, and *A. butzleri* UPa 2015/14. A significant decrease in biofilm formation of these strains was observed even at the lowest concentration of this antibiotic. The minimum inhibitory concentration of ciprofloxacin to all the above strains reached values of 0.06 mg/L. Probably due to the significant inhibitory effect of ciprofloxacin on these arcobacterial strains, they were completely devitalized, even at low concentrations. Therefore, ciprofloxacin was evaluated as the most effective antibiotic for suppressing biofilm formation.

## 3. Discussion

The increasing incidence of antimicrobial-resistant bacteria is at the forefront of scientific research. Monitoring and reporting antimicrobial resistance is important, and the aim is to emphasize the threat of *Arcobacter*-like microorganisms and other microorganisms [18]. The extensive use of antibiotics could lead to the development and spread of antibiotic-resistant strains around the world. The aim of this study was to provide information about the antimicrobial effect of common antibiotics used in human and veterinary medicine for the inhibition of *Arcobacter*-like microorganisms. It has already been noted that antibiotic susceptibility testing for arcobacters has not been standardized yet [22]. Therefore, it is difficult to compare results from different studies; nevertheless, the methodology is usually based on EUCAST or CLSI breakpoints [19,20,21].

Illness caused by *Arcobacter*-like strains can be treated with antibiotics; however, the individual strains can considerably differ in their sensitivity to antibiotics. Generally, a lot of studies recommend tetracyclines and aminoglycosides as first-line antibiotics in the treatment of arcobacteriosis [21,25,26]. Ferreira et al. reported that none of the tested strains exhibited resistance to gentamicin or tetracycline [27]. Our results agreed with the zero resistance of arcobacters to gentamicin; however, in our study it was found that 65.0% of the tested strains were resistant to tetracycline. A majority of studies report high susceptibility of arcobacters to tetracycline (and suggest the use of tetracycline for treatment of human and animal infections); however, our study demonstrates an increasing resistance of the isolates. Some previous studies also report resistance of arcobacters to tetracycline [18]. According to our results, ciprofloxacin (quinolones) could be used for the treatment of arcobacteriosis. The susceptibility of arcobacters to ciprofloxacin was also reported by other studies [28,29,30]. Overall, the lowest levels of resistance were observed to erythromycin, gentamicin, and ciprofloxacin, which is in accordance with previously reported data [25,28,29,31]. Several studies have also reported multi-drug resistance to antibiotics that basically corresponds to our results [19,21]. However, the designations of multi-resistant strains vary among studies.

Due to insufficient information of the effect of antibiotics on biofilm formation in the literature, several collection strains (*A. butzleri* CCUG 30484, *A. cryaerophilus* CCM 7050, *Pseudarcobacter defluvii* LMG 25694, *A. skirrowii* LMG 6621) and isolates obtained within the Czech Republic were included in our study. It is well known that biofilm cells can be a significant source of infection. It is also necessary to consider the many-fold increased resistance of microorganisms to many antibiotics and other antimicrobial substances [15]. Bacteria in biofilm structures are up to 1000 times more resistant to antibiotics than planktonic cells. It follows that the MIC values determined for planktonic cells are not applicable for estimating the effect of antibiotics on biofilm formation, where a need for a higher concentration can logically be assumed [32]. Biofilm formation has also been previously described in *Arcobacter*-like microorganisms [8,14,29,33]. The initial biofilm formation activities of the studied strains were different. The influence of the environment is not only fundamental to the planktonic cells of microorganisms, but also for biofilm formation [34]. In the literature, *Arcobacter*-like strains are generally described as weak biofilm producers [14,16,35]. However, our results show that some arcobacters are capable of intensive biofilm formation, even in the presence of antibiotics (e.g., *Pseudarcobacter defluvii* LMG 25694; see Figure 2). This fact could be explained by the higher biofilm formation in response to stress [16]. In contrast, many studies have shown that antibiotics at sub-MIC concentrations can significantly induce biofilm formation in a variety of bacterial strains in vitro [32]. Enhancement of biofilm formation at sub-MIC concentrations of antibiotics is probably a useful strategy of pathogenic bacteria facilitating their survival after intense antibiotic therapy when low concentrations of drugs remain in the human organism [36,37]. Although the strains are sensitive to antibiotics, they may have a high resistance due to their biofilm formation ability. Thus, caution should be exercised in setting the correct therapeutic dose of antibiotics and keeping in mind that biofilm formation allows some microorganisms to escape the effects of some antibiotics. There is no single mechanism of antibiotic-induced biofilm formation; however, cellular stress seems to play a role in many bacteria. A full understanding of this process can help lead to the development of new antibiotic substances that would suppress biofilm formation. It has been reported that the use of tetracycline may lead to increased biofilm formation [38]. However, this assumption was not confirmed in our study (only for some strains at the lowest concentrations). Based on the obtained results, it is assumed that biofilm formation is supported by the presence of antibiotics at sub-MIC concentrations, in agreement with a previous study [39]. On the other hand, for some microorganisms, it has also been reported in the past that even sub-MIC concentrations of antibiotics lead to an inhibition of biofilm formation [38]. Furthermore, it was previously described that ciprofloxacin, amikacin, and colistin reduced the biofilm formation of *E. coli*, so these antibiotics could be useful in the treatment of biofilm-associated infections caused by similar strains [40]. It was also confirmed that the lowest concentration of fluoroquinolones caused a reduction of the initial biofilm [15]. According to our results, ciprofloxacin was the most effective antibiotic at eliminating *Arcobacter*-like biofilm formation ability, even at lower concentrations. Different strategies need to be combined to improve the antimicrobial effectiveness of antimicrobial substances, due to the multi-species form of the biofilm environment and increased tolerance to antibiotics or antimicrobial substances [41].

## 4. Materials and Methods

### 4.1. Arcobacter-like Strains

A total of 60 strains of *Arcobacter*-like microorganisms were used for antibiotic susceptibility testing and for determining the effect of antibiotics on their biofilm activity. Strains were obtained from the Czech Collection of Microorganisms (CCM, Brno, Czech Republic), Culture Collection University of Göteborg (CCUG, Göteborg, Sweden), Belgian Co-ordinated Collections of Microorganisms (LMG, Ghent, Belgium) or isolated from food of animal origin (*n* = 34), water (*n* = 16) and clinical samples (*n* = 3) at the University of Pardubice (UPa, Pardubice, Czech Republic).

Cultures were grown on Tryptone Soya agar (TSA, HiMedia, Mumbai, India) for 48 h at 30 °C before testing. Cells were suspended in physiological saline to a value of 0.5 on the McFarland scale (3–9 × 10^8^ CFU/mL). The suspension of cells was then diluted to an appropriate density before each testing.

### 4.2. Antibiotic Susceptibility Testing

Minimum inhibitory concentration (MIC) and minimum bactericidal concentration (MBC) were determined by a microdilution method in 96–well microtiter plates (SPL Life Sciences, Pocheon-si, South Korea). Ampicillin (AMP), ciprofloxacin (CIP), clindamycin (CLI), erythromycin (ERY), gentamicin (GEN) and tetracycline (TET) purchased from Sigma-Aldrich (St. Louis, MO, USA) were used for testing. Stock solution of antibiotics at a concentration of 1024 mg/L were stored according to the manufacturer’s instructions at 2–8 °C or −20 °C until use. The antibiotic lines (0.03–256 mg/L) for testing were prepared in Mueller–Hinton broth II (MH-II, Merck, Germany) in accordance with a previous study and recommendation [22]. Each well (total volume of 100 µL) was inoculated with a cell suspension to obtain a final cell density of 1.5 × 10^6^ CFU/mL. After 24 h of cultivation at 30 °C and aerobic conditions, the content of each well was sub-cultured onto nonselective TSA medium. After cultivation, MIC was recorded as the lowest concentration of the antibiotic that inhibited the visible growth of microorganisms (99.9%). MIC_50_ and MIC_90_ indicate the concentration at which the tested isolates were visibly inhibited by the antibiotics at the level of 50% or 90%, respectively. The bactericidal activity (MBC) was evaluated as the lowest concentration of antibiotics needed to kill 99.9% of cells after sub-culturing a sample from wells. Each experiment was performed at least four times. For the *Arcobacter*-like microorganisms, no recommendations of breakpoint values are available. Strains were classified based on the breakpoints recommended for the closely related *Campylobacter coli* (erythromycin, ciprofloxacin, and tetracycline) and family *Enterobacteriaceae* according to the Clinical Laboratory Standards Institute and in accordance with a previous study [18,24].

### 4.3. Biofilm Formation of Arcobacter-like Strains in the Presence of Antibiotics

The effect of different concentrations of the antibiotics on biofilm formation was monitored in 96-well polystyrene flat-bottomed microtiter plates (SPL Life Sciences, Pocheon-si, South Korea) as previously described [42]. Briefly, the two-fold dilutions of antibiotics were prepared in brain heart infusion broth (BHI, HiMedia, India) to obtain a final concentration ranging from 0.03 to 128 mg/L in the wells after the addition of 10 µL of the freshly diluted cell culture at a cell density of 10^8^ CFU/mL (final volume 100 µL). After incubation at 30 °C for 24 h under aerobic conditions, the microtiter plate was repeatedly washed with sterile distilled water and dried. Biofilm fixation was performed with 2% sodium acetate (15 min) and biofilm-forming cells were stained with 100 µL of 1% crystal violet (Sigma-Aldrich, St. Louis, MO, USA). After 15 min of staining, the plate was repeatedly washed and dried. Thereafter, the biofilm-associated violet was solubilized with 96% ethanol and the optical density of the solution was measured in a new plate at 595 nm (Infinite M200, Tecan, Männedorf, Switzerland). There were 8 replicate wells in each experiment, experiments were independently repeated 3 times. The biofilm formation level of the *Arcobacter*-like strains was categorized according to a previously described classification system [16] as non-adherent (OD ≤ OD_C_) or biofilm-forming strains (OD > OD_C_), where ODC (cut-off OD) is defined as three standard deviations above the mean OD of the negative control (blank value). The measured and calculated OD/OD_C_ (0.111/0.120) values were the same for all measurements.

### 4.4. Statistical Analysis

The obtained values were statistically evaluated using Excel 2010 (Microsoft, Redmond, WA, USA) and Statistica 12 (StatSoft, Tulsa, OK, USA). Extreme values were tested with the Dean–Dixon test, and all remoteness values were excluded with 95% probability. Median and standard deviations were determined from the remaining values. A possible source of error resulting in an increase in absorbance was also considered, and absorbance values that were too high compared to other measured values were excluded.

## 5. Conclusions

In conclusion, this study is to our knowledge the first report to assess the antibiotic susceptibility of less described collection strains of arcobacters, and also of strains isolated within the Czech Republic. This study showed the diversity of responses to antibiotic treatment in *Arcobacter*-like strains depending on their planktonic or biofilm form. The study also provides an important insight into the ability of many strains to form a biofilm. Although many strains were evaluated as being the most sensitive to ciprofloxacin, gentamicin, and erythromycin, biofilm formation was observed at antibiotic concentrations below the MICs. In general, antibiotic concentrations around their MIC or sub-MIC values are dangerous in terms of the increased biofilm formation of these bacteria. It is concluded that biofilm-forming bacteria are able to avoid the effects of commonly used antibiotics. Knowledge about the consequences of antimicrobial agents and treatment of bacterial infections needs to be examined and appropriate concentrations need to be selected, taking into account the biofilm structure [32]. It is important to monitor the biofilm formation of these bacteria, because they are responsible for an influential percentage of human alimentary infections. Subsequent studies of the influence of antibiotics on planktonic cells would have great benefits for the food industry and healthcare. Moreover, since biofilm formation is a serious problem for antimicrobial therapy, special attention should be paid to the effect of drugs in medical practice.

## Figures and Tables

**Table 1:** Minimum inhibitory concentration (MIC) and identical minimum bactericidal concentration (MBC) data on antibiotic agents determined for 60 *Arcobacter*-like strains isolated within the Czech Republic and collection strains.

	Number of Isolates Susceptible at Given Concentrations and MICs (mg/L)																
	0.03	0.06	0.13	0.25	0.5	1	2	4	8	16	32	64	128	256	MIC_50_	MIC_90_	R
**Ampicillin**																	
*A. butzleri* strains (50)			1	3	2	1	4	5	15	7	6	5	1		8	64	24%
*A. cryaerophilus* strains (3)									1	2					8	128	0%
*Ab* CCUG 30484										1							
*Ab* LMG 10828													1				
*Ac* CCM 3933										1							
*Ac* CCM 3934								1									
*Ac* CCM 7050								1									
*Pd* LMG 25694								1									
*As* LMG 6621										1							
**Ciprofloxacin**																	
*A. butzleri* strains (50)	20	13	12	3							1	1			0.06	0.13	4%
*A. cryaerophilus* strains (3)	1		1									1			0.13	64	33.3%
*Ab* CCUG 30484		1															
*Ab* LMG 10828			1														
*Ac* CCM 3933		1															
*Ac* CCM 3934		1															
*Ac* CCM 7050			1														
*Pd* LMG 25694		1															
*As* LMG 6621		1															
**Clindamycin**																	
*A. butzleri* strains (50)		2		1	3	1	2	2	5	7	15	6	6		32	128	86%
*A. cryaerophilus* strains (3)								1		1	1				16	32	100%
*Ab* CCUG 30484												1					
*Ab* LMG 10828														1			
*Ac* CCM 3933									1								
*Ac* CCM 3934										1							
*Ac* CCM 7050												1					
*Pd* LMG 25694								1									
*As* LMG 6621								1									
**Erythromycin**																	
*A. butzleri* strains (50)		1		3	4	8	13	17	4						2	4	0%
*A. cryaerophilus* strains (3)						1	1	1							2	4	0%
*Ab* CCUG 30484					1												
*Ab* LMG 10828									1								
*Ac* CCM 3933							1										
*Ac* CCM 3934							1										
*Ac* CCM 7050							1										
*Pd* LMG 25694						1											
*As* LMG 6621				1													
**Gentamicin**																	
*A. butzleri* strains (50)		12		22	9	6	1								0.25	1	0%
*A. cryaerophilus* strains (3)				1	1	1									0.5	1	0%
*Ab* CCUG 30484					1												
*Ab* LMG 10828									1								
*Ac* CCM 3933						1											
*Ac* CCM 3934				1													
*Ac* CCM 7050				1													
*Pd* LMG 25694		1															
*As* LMG 6621					1												
**Tetracycline**																	
*A. butzleri* strains (50)				3	2	8	10	7	5	7	4	1	3		4	64	54%
*A. cryaerophilus* strains (3)							1			1	1				16	32	66.7%
*Ab* CCUG 30484													1				
*Ab* LMG 10828													1				
*Ac* CCM 3933												1					
*Ac* CCM 3934									1								
*Ac* CCM 7050									1								
*Pd* LMG 25694										1							
*As* LMG 6621										1							

*A.—Aliarcobacter; Ab—A. butzleri; Ac—A. cryaerophilus; Pd—Pseudarcobacter defluvii; As—A. skirrowii.* Resistance breakpoints were applied according to the Clinical and Laboratory Standards Institute established for *C. coli* and family *Enterobacteriaceae* [24]. Grey shading indicates resistant strains. R—percentage of resistant strains.

**Table 2:** Multi-drug resistance of *Arcobacter*-like strains.

	Resistance Patterns						
*Arcobacter*-like Strain	AMP, CLI	AMP, TET	CLI, TET	AMP, CLI, TET	CIP, CLI, TET	AMP, CIP, CLI, TET	AMP, CLI, GEN, TET
*A. butzleri* strains (50)	3	1	17	6	1	1	
*A. cryaerophilus* strains (3)				1	1		
*Ab* CCUG 30484			1				
*Ab* LMG 10828							1
*Ac* CCM 3933			1				
*Ac* CCM 3934			1				
*Ac* CCM 7050			1				
*Pd* LMG 25694			1				
*As* LMG 6621			1				

*A.*—*Aliarcobacter*; *Ab*—*A. butzleri*; *Ac*—*A. cryaerophilus*; *Pd*—*Pseudarcobacter defluvii*; *As*—*A. skirrowii*. AMP—ampicillin; CIP—ciprofloxacin; CLI—clindamycin; GEN—gentamicin; TET—tetracycline. Multi-drug resistance was defined as resistance to two or more of the tested antibiotics.

## References

[1] Vandamme P., Falsen E., Rossau R., Hoste B., Segers P., Tytgat R., De Ley J. (1991). Revision of *Campylobacter*, *Helicobacter*, and *Wolinella* taxonomy: Emendation of generic descriptions and proposal of *Arcobacter* gen. nov. Int. J. Syst. Bacteriol..

[2] Schumacher W., Kroneck P.M.H., Pfennig N. (1992). Comparative systematic study on Spirillum 5175, *Campylobacter* and *Wolinella* species: Description of *Spirillum* 5175 as *Sulfurospirillum deleyianum* gen. nov., spec. nov. Arch. Microbiol..

[3] Chieffi D., Fanelli F., Fusco F. (2020). *Arcobacter butzleri*: Up-to-date taxonomy, ecology, and pathogenicity of an emerging pathogen. Compr. Rev. Food Sci. Food Saf..

[4] Waite D.W., Vanwonterghem I., Rinke C., Parks D.H., Zhang Y., Takai K., Hugenholtz P. (2017). Comparative genomic analysis of the class *Epsilonproteobacteria* and proposed reclassification to *Epsilonbacteraeota* (phyl. nov.). Front. Microbiol..

[5] Waite D.W., Vanwonterghem I., Rinke C., Parks D.H., Zhang Y., Takai K., Hugenholtz P. (2018). Addendum: Comparative genomic analysis of the class *Epsilonproteobacteria* and proposed reclassification to *Epsilonbacteraeota* (phyl. nov.). Front. Microbiol..

[6] Ferreira S., Oleastro M., Domingues F. (2019). Current insights on *Arcobacter butzleri* in food chain. Curr. Opin. Food Sci..

[7] Debruyne L., Gevers D., Vandamme P. (2008). Taxonomy of the Family Campylobacteraceae Campylobacter.

[8] Šilha D., Hrušková L., Brožková I., Moťková P., Vytřasová J. (2014). Survival of selected bacteria from the genus *Arcobacter* on various metallic surfaces. J. Food Nutr. Res..

[9] Fanelli F., Pinto Di A., Mottola A., Mule G., Chieffi D., Baruzzi F., Fusco V. (2019). Genomic characterization of *Arcobacter butzleri* isolated from shellfish: Novel insight into antibiotic resistance and virulence determinants. Front. Microbiol..

[10] Šilha D., Vackova B., Šilhova L. (2019). Occurrence of virulence-associated genes in *Arcobacter butzleri* and *Arcobacter cryaerophilus* isolates from foodstuff, water, and clinical samples within the Czech Republic. Folia Microbiol..

[11] Collado L., Figueras M.J. (2011). Taxonomy, Epidemiology, and Clinical Relevance of the Genus *Arcobacter*. Clin. Microbiol. Rev..

[12] Ferreira S., Queiroz J.A., Oleastro M., Domingues F.C. (2016). Insights in the pathogenesis and resistance of *Arcobacter*: A review. Crit. Rev. Microbiol..

[13] Miller W.G., Parker C.T., Rubenfield M., Mendz G.L., Wosten M.M., Ussery D.W., Mandrell R.E. (2007). The complete genome sequence and analysis of the epsilonproteobacterium *Arcobacter butzleri*. PLoS ONE.

[14] Girbau C., Martinez-Malaxetxebarria I., Muruaga G., Carmona S., Alonso R., Fernandez-Astorga A. (2017). Study of biofilm formation ability of foodborne *Arcobacter butzleri* under different conditions. J. Food Protect..

[15] Passerini de Rossi B., García C., Calenda M., Vay C., Franco M. (2009). Activity of levofloxacin and ciprofloxacin on biofilms and planktonic cells of *Stenotrophomonas maltophilia* isolates from patients with device-associated infections. Int. J. Antimicrob. Agents.

[16] Šilha D., Sirotková S., Švarcová K., Hofmeisterová L., Koryčanová K., Šilhová L. (2021). Biofilm Formation Ability of *Arcobacter*-Like and *Campylobacter* Strains under Different Conditions and on Food Processing Materials. Microorganisms.

[17] Ramees T.P., Dhama K., Karthik K., Rathore R.S., Kumar A., Saminathan M., Tiwari R., Malik Y.S., Singh R.K. (2017). *Arcobacter*: An emerging food-borne zoonotic pathogen, its public health concerns and advances in diagnosis and control—A comprehensive review. Vet. Q..

[18] Van den Abeele V., Vogelaers D., Vanlaere E., Houf K. (2016). Antimicrobial susceptibility testing of *Arcobacter butzleri* and *Arcobacter cryaerophilus* strains isolated from Belgian patients. J. Antimicrob. Chem..

[19] Šilha D., Pejchalova M., Šilhova L. (2017). Susceptibility to 18 drugs and multidrug resistance of *Arcobacter* isolates from different sources within the Czech Republic. J. Glob. Antimicrob. Resist..

[20] Vicente-Martins S., Oleastro M., Domingues F.C., Ferreira S. (2018). *Arcobacter* spp. at retail food from Portugal: Prevalence, genotyping and antibiotics resistance. Food Control.

[21] Rathlavath S., Kohli V., Singh A.S., Lekshmi M., Tripathi G., Kumar S., Nayak B.B. (2017). Virulence genotypes and antimicrobial susceptibility patterns of *Arcobacter butzleri* isolated from seafood and its environment. Int. J. Food Microbiol..

[22] Riesenberg A., Frömke C., Stingl K., Feßler A.T., Gölz G., Glocker E.O., Kreienbrock L., Klarmann D., Werckenthin C., Schwarz S. (2017). Antimicrobial susceptibility testing of *Arcobacter butzleri*: Development and application of a new protocol for broth microdilution. J. Antimicrob. Chem..

[23] Zacharow I., Bystroń J., Wałecka-Zacharska E., Podkowik M., Bania J. (2015). Prevalence and antimicrobial resistance of *Arcobacter butzleri* and *Arcobacter cryaerophilus* isolates from retail meat in Lower Silesia region, Poland. Pol. J. Vet. Sci..

[24] Clinical and Laboratory Standards Institute (CLSI) (2017). Performance Standards for Antimicrobial Susceptibility Testing.

[25] Aski S., Tabatabaei H., Khoshbakht M., Raeisi M. (2016). Occurrence and antimicrobial resistance of emergent *Arcobacter* spp. isolated from cattle and sheep in Iran. Comp. Immunol. Microbiol. Infect. Dis..

[26] Kayman T., Abay S., Hizlisoy H., Atabay H.İ., Diker K.S., Aydin F. (2012). Emerging pathogen *Arcobacter* spp. in acute gastroenteritis: Molecular identification, antibiotic susceptibilities and genotyping of the isolated arcobacters. J. Med. Microbiol..

[27] Ferreira S., Queiroz J.A., Oleastro M., Domingues F.C. (2014). Genotypic and phenotypic features of *Arcobacter butzleri* pathogenicity. Microb. Pathog..

[28] Vandenberg O., Houf K., Douat N., Vlaes L., Retore P., Butzler J.P., Dediste A. (2006). An-timicrobial susceptibility of clinical isolates of non-*jejuni/coli* campylobacters and arcobacters from Belgium. J. Antimicrob. Chem..

[29] Ferreira S., Fraqueza M.J., Queiroz J.A., Domingues F.C., Oleastro M. (2013). Genetic diversity, antibiotic resistance and biofilm-forming ability of *A. butzleri* isolated from poultry and environment from a Portuguese slaughterhouse. Int. J. Food Microbiol..

[30] Houf K., Devriese L.A., Haesebrouck F., Vandenberg O., Butzler J.P., van Hoof J., Vandamme P. (2004). Antimicrobial susceptibility patterns of *Arcobacter butzleri* and *Arcobacter cryaerophilus* strains isolated from humans and broilers. Microb. Drug Resist..

[31] Abay S., Kayman T., Hizlisoy H., Fuat A. (2011). In vitro Antibacterial Susceptibility of *Arcobacter butzleri* Isolated from Different Sources. J. Vet. Med. Sci..

[32] Tezel U.B., Akcelik N., Yuksel F.N., Karatuğ N., Akcelik M. (2016). Effects of sub-MIC antibiotic concentrations on biofilm production of *Salmonella* Infantis. Biotechnol. Biotechnolog. Equip..

[33] Kjeldgaard J., Jørgensen K., Ingmer H. (2009). Growth and survival at chiller temperatures of *Arcobacter butzleri*. Int. J. Food Microbiol..

[34] Jabra-Rizk M.A., Meiller T.F., James C.E., Shirtli M.E. (2006). Effect of farnesol on *Staphylococcus aureus* biofilm formation and antimicrobial susceptibility. Antimicrob. Agents Chem..

[35] Šilhová-Hrušková L., Moťková P., Šilha D., Vytřasová J. (2015). Hodnocení tvorby biofilmu vybraných patogenů vyskytujících se v potravinářském průmyslu. Epidemiol. Mikrobiol. Imunol..

[36] Costerton J.W., Stewart P.S., Greenberg E.P. (1999). Bacterial biofilms: A common cause of persistent infections. Science.

[37] Plyuta V., Zaitseva J., Lobakova E., Zagoskina N., Kuznetsov A., Khmel I. (2013). Effect of plant phenolic compounds on biofilm formation by *Pseudomonas aeruginosa*. APMIS.

[38] Kaplan J.B. (2011). Antibiotic-induced biofilm formation. Int. J. Artif. Org..

[39] Duarte A., Alves A.C., Ferreira S., Silva F., Domingues F.C. (2015). Resveratrol inclusion complexes: Antibacterial and anti-biofilm activity against *Campylobacter* spp. and *Arcobacter butzleri*. Food Res. Int..

[40] Wojnicz D., Tichaczek-Goska D. (2013). Effect of sub-minimum inhibitory concentrations of ciprofloxacin, amikacin and colistin on biofilm formation and virulence factors of Escherichia coli planktonic and biofilm forms isolated from human urine. Braz. J. Microbiol..

[41] Ciofu O., Rojo-Molinero E., Macia M.D., Oliver A. (2017). Antibiotic treatment of biofilm infections. APMIS.

[42] Šilha D., Švarcova K., Bajer T., Královec K., Tesarova E., Mouckova K., Pejchalová M., Bajerova P. (2020). Chemical Composition of Natural Hydrolates and Their Antimicrobial Activity on *Arcobacter*-Like Cells in Comparison with Other Microorganisms. Molecules.

